# Supplementary material for: Genuine quantum scars in many-body spin systems
Source: Nat Commun. 2025 Jul 21;16:6722. doi: 10.1038/s41467-025-61765-3 (PMC12280054; doi:10.1038/s41467-025-61765-3)
Supplement: Supplementary file 1 — Supplementary Information [file 41467_2025_61765_MOESM1_ESM.pdf]

Supplementary Information for
"Genuine quantum scars in many-body spin systems"

Andrea Pizzi, Long-Hei Kwan, Bertrand Evrard, Ceren B. Dag, and Johannes Knolle

This Supplementary Information is devoted to technical derivations and complementary details. It is structured as follows. In Section I, we present some complementary results supporting the ubiquity of quantum scarring in many-body systems. In Section II we develop the tools needed to compute the Lyapunov exponent of the IS states, namely we perform a zero-th order Floquet expansion in a frame precessing around the external magnetic field $\boldsymbol{\mu}$. In Section III we compute the Lyapunov exponent for the IS UPOs in the limit of small $|\mathbf{J}|/|\boldsymbol{\mu}|$. In Section IV we provide a few more details on computing classical phase-space projections.

I) UBIQUITY OF SCARS

We aim here to solidify the claim that scarring is ubiquitous in many-body spin chains, affecting many eigenstates across various models.

Qualitative, visual features of scars

First, in Fig. S1 we generalize Fig. 2 from the main text, considering the Ising, XXZ, and XX models in a field. Moreover, we do not consider a selection of eigenstates, but simply the 10 eigenstates in the middle of the spectrum (that is composed of 8356 eigenstates overall). The Ising model is like that in the main text, the XXZ model reads

$$\hat{H} = \frac{1}{2}\sum_j \left[\boldsymbol{\mu}\cdot\hat{\boldsymbol{\sigma}}_j + J_{xx}\left(\hat{\sigma}_j^x\hat{\sigma}_{j+1}^x + \hat{\sigma}_j^y\hat{\sigma}_{j+1}^y\right) + J_{zz}\hat{\sigma}_j^z\hat{\sigma}_{j+1}^z\right], \tag{S1}$$

and the XX model reads

$$\hat{H} = \frac{1}{2}\sum_j \left[\boldsymbol{\mu}\cdot\hat{\boldsymbol{\sigma}}_j + J_{xx}\left(\hat{\sigma}_j^x\hat{\sigma}_{j+1}^x + \hat{\sigma}_j^y\hat{\sigma}_{j+1}^y\right)\right]. \tag{S2}$$

The specific values of the parameters are the same as in Fig. 2 in the main text, and ensure that the system is well thermalized. The expectation value $\langle E_n|\sigma_1^x|E_n\rangle$ and the bipartite entanglement entropy S for the eigenstates $|E_n\rangle$ is shown in Fig. S1, and confirms thermalization. The projection of the 10 central eigenstates onto the manifold of IS UPOs is shown in Fig. S1(b). To various extents, for the majority of the eigenstates the projection Q reflects the underlying UPOs. More mid-spectrum eigenstates are shown, for the Ising model, in Fig. S1(c). By contrast, in Fig. S2 we see that the projection Q of Haar random states does not reflect in a any way the structure of the UPOs (indeed, the random states are agnostic of the Hamiltonian, and hence of the UPOs).

Quantitative features of scarring: details and role of the symmetries

We now turn to the question how to quantify scarring, and in particular whether the eigenstates have an anomalously large projection Q on the UPOs. This is nontrivial, because the meaning of "large" is subtle: just out of random fluctuations, even the projection of a Haar random wavefunction happens to be somewhat larger in some points of the phase space, as in Fig. S2, and indeed have some finite probability to be arbitrarily close to the maximum value 1. In what sense then, exactly, can the projection of a scarred eigenstate on a UPO be said "anomalously large"?

The first and mandatory step to sensibly address these questions is to analyze the symmetries of the problem. The system is invariant under translation, which splits the Hamiltonian in sectors with a well defined momentum $k = \frac{2\pi}{N}n$, with $n = 0, 1, 2, \dots N-1$. Due to the left-right mirror reflection symmetry, the sectors with $k=0$ and $k=\pi$ can be further divided into blocks with parity $P=\pm 1$. The symmetry sectors can thus be tagged by their momentum k and, if $k=0$ or $k=\pi$, by a superscript $\pm$ indicating the parity. Let us call $\hat{\mathcal{P}}_\nu$ the operator that projects from the full Hilbert space to the ν-th sector. We denote $\mathcal{D}_\nu$ the size of the ν-th sector, and $\mathcal{D} = \sum_\nu \mathcal{D}_\nu$ the total size of the Hilbert space. For instance, for $s=1/2$ and $N=20$ we find $\mathcal{D}_{0^+} = 27012$, $\mathcal{D}_{\pi^+} = 25984$, $\mathcal{D}_{0^-} = 25476$, $\mathcal{D}_{\pi^-} = 26496$, $\mathcal{D}_{\pi/2} = 52380$, and $\mathcal{D} = 1048576$.

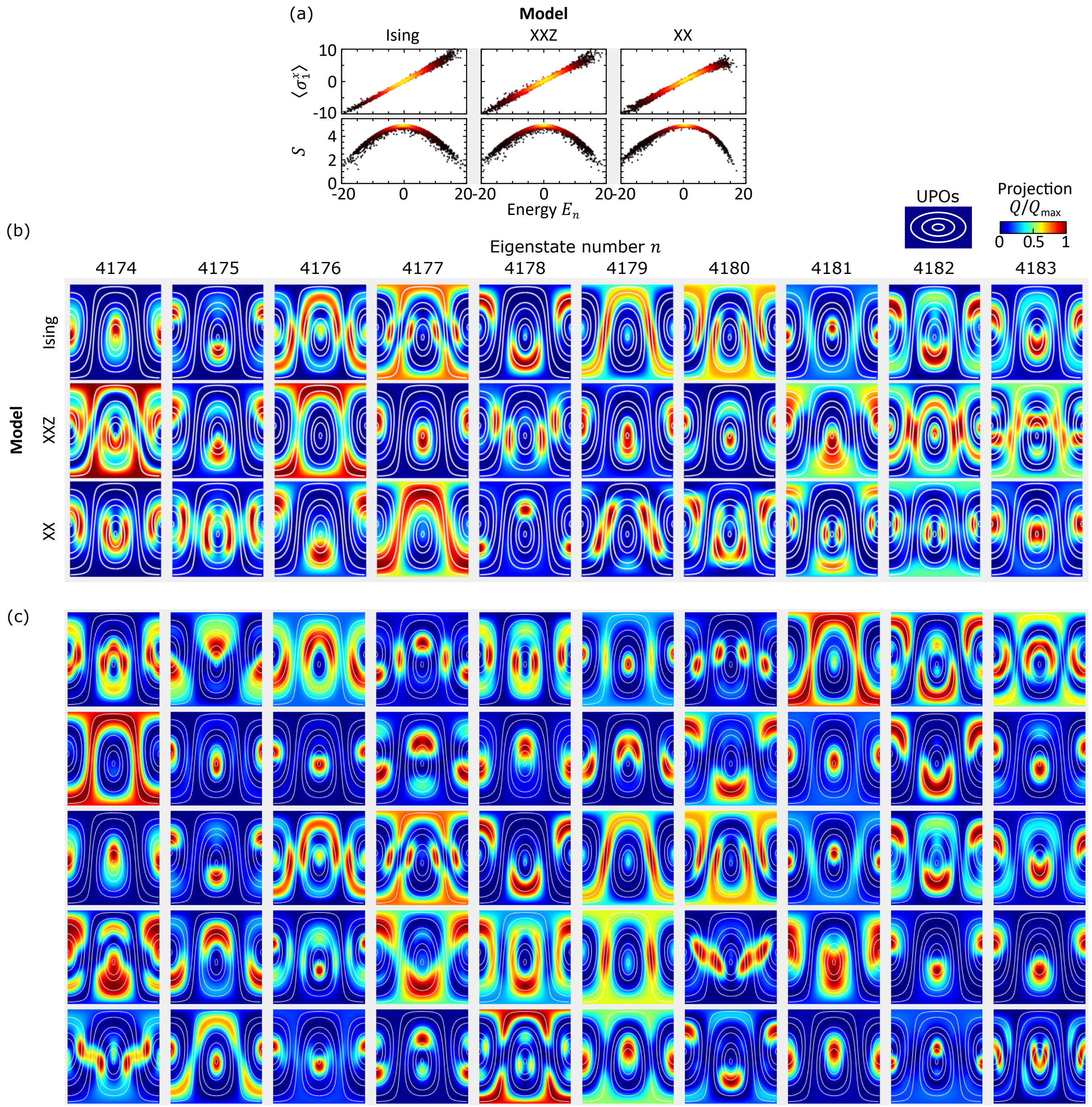

Fig. S1. **Ubiquity of quantum scars.** (a) Expectation value $\langle E_n|\sigma_1^x|E_n\rangle$ and bipartite entanglement entropy S for the eigenstates $|E_n\rangle$ of the Ising, XXZ, and XX models. (b) Projection over the manifold of IS states of 10 consecutive eigenstates taken in the middle of the spectrum. Most of the eigenstates appear scarred, to some variable extent, by the UPOs. (c) Same as in (b), but for 50 mid-spectrum eigenstates and specifically for the Ising model. The eigenstate number n runs along the rows from 4154 in the top left to 4203 in the bottom right. Here, $N = 16$.

It is easy to verify that the TI states are fully contained within the 0^+ sector while the IS states distribute their weight equally within four "special" sectors 0^+, π^+, $+\frac{\pi}{2}$, and $-\frac{\pi}{2}$. It follows that one can talk about scarring from the TI UPOs only for the eigenstates of the sector 0^+, and scarring from the IS UPOs only for the eigenstates of the sectors 0^+, π^+, $+\frac{\pi}{2}$, and $-\frac{\pi}{2}$. This is why in Fig. 2 in the main text we considered the eigenstates of the four special sectors, while in Fig. 2(c) we considered eigenstates of the sector 0^+ (the only sector that can be scarred by the TI and IS states at the same time).

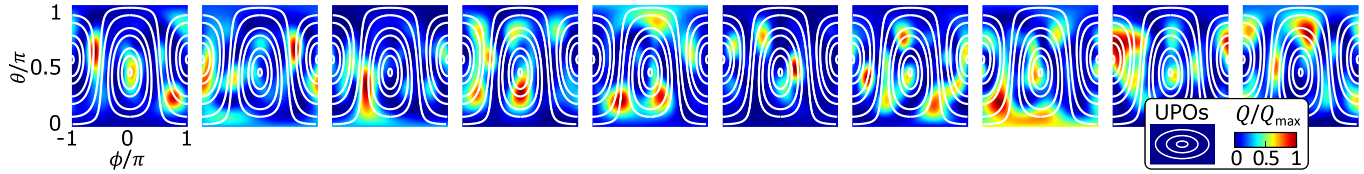

Fig. S2. **Phase-space projection of Haar random states.** Projection Q of Haar random states, for $N = 16$. The random states do not show any noteworthy structure in relation to the underlying classical UPOs.

These symmetry considerations done, let us go back to the question whether the projection of the eigenstates on the UPOs is "large", focussing on one of the four special sectors. We consider the 10% most central (i.e., with smallest $|E_n|$) eigenstates of the sector. Let us call M_e the number of such mid-spectrum eigenstates, for instance $M_e = 2701$ for $s = 1/2$, $N = 20$, and sector $\nu = 0^+$. As in Fig. 3 in the main text, to quantify scarring we want to compare the overlap of the mid-spectrum eigenstates with various families of states. The first are Haar random states, that should be sampled within the symmetry sector of interest. The second are generic phase space states $|\{\mathbf{s}_i\}\rangle$, drawn upon sampling the spins $\{\mathbf{s}_i\}$ uniformly and independently from the surface of a unit sphere. The third and last are IS states, which are also of the form $|\{\mathbf{s}_i\}\rangle$, but for which $\mathbf{s}_1$ is sampled uniformly from the surface of a sphere while all the other spins are set following the IS condition, namely $\mathbf{s}_i = \nu_i \mathbf{s}_1$ with $\nu_i = (++--++--\dots)$. Crucially, generic phase-space states $|\{\mathbf{s}_i\}\rangle$ lack any symmetry, and their weight is thus distributed among all symmetry sectors, e.g., only $\approx 1/(2N)$-th of their weight is on the 0^+ sector on average. By contrast, the IS states are highly symmetrical and have a relatively large weight $1/4$ on each of the special symmetry sectors. Just out of symmetry arguments it follows that an eigenstate of one of the special sectors will tend to have a larger overlap with an IS state than with a generic phase space state, on average by a factor $\sim \frac{1/4}{1/(2N)} = \frac{N}{2}$ for sectors 0^+ and π^+, and by a factor $\sim \frac{1/4}{1/(N)} = \frac{N}{4}$ for sectors $+\frac{\pi}{2}$ and $-\frac{\pi}{2}$. Of course, such an enhancement is first of all an effect of the symmetries, not of scarring.

To distill the enhancement due to scarring, we effectively cancel the effect of the symmetries by projecting out the part of the wavefunction that is not in the symmetry sector of interest. That is, given a state $|\psi\rangle$ (e.g., a phase-space state or a IS state), we define the state

$$|\psi\rangle_\nu = \frac{\hat{\mathcal{P}}_\nu^\dagger |\psi\rangle}{\sqrt{\langle\psi| \hat{\mathcal{P}}_\nu \hat{\mathcal{P}}_\nu^\dagger |\psi\rangle}}, \tag{S3}$$

that, by construction, has weight 1 on the symmetry sector of interest ν, and 0 on the other sectors. Thus, while phase-space states and IS states have on average a different weight on ν, their projected versions in Eq. (S3) by construction have the same weight 1, and can thus be compared fairly. Furthermore, now that all the states of interest have weight 1 on the considered sector ν, we can also conclude that their average weight on the basis states of such sector is $\mathcal{D}_\nu^{-1}$. For a sector of interest $\nu \in (0^+, \pi^+, +\frac{\pi}{2}, -\frac{\pi}{2})$, an eigenstate $|E\rangle_n$ of such sector, and a state $|\psi\rangle$, we can thus define the rescaled overlap

$$x = \mathcal{D}_\nu \left|\langle E_n|\psi\rangle_\nu\right|^2 = \mathcal{D}_\nu \frac{\left|\langle E_n|\psi\rangle\right|^2}{\langle\psi|\hat{\mathcal{P}}_\nu \hat{\mathcal{P}}_\nu^\dagger|\psi\rangle}, \tag{S4}$$

that is what we have used in the main text for the case of $\nu = 0^+$. Specifically, to quantify the overlap of the eigenstates with a certain family of states (Haar, phase-space states, or IS states), we generate an ensemble of rescaled projections x upon sampling both $|E_n\rangle$ (from the mid-spectrum eigenstates) and $|\psi\rangle$ (from the specific family of states). For each family of states this results in a probability distribution for x, which we have plotted in Fig. 3.

As mentioned above, scarring manifests in Q having large fluctuations and having a structure in phase space that reflects the underlying UPOs. To quantify both effects at once, and focusing on the IS case, we consider the average Q over an IS orbit, which we denote $\oint_{\mathrm{IS}} Q$, and look for the IS orbit that maximizes it. For a wavefunction $|\psi\rangle$ with projection Q we thus introduce the following figure of merit for scarring:

$$S = 4\mathcal{D}_\nu \times \max_{\text{IS orbits}} \oint_{\mathrm{IS}} Q, \tag{S5}$$

where $\mathcal{D}_\nu$ is the size of the considered special symmetry sector and the factor 4 compensates for the fact that the IS states have weight $1/4$ on it (these normalization factors are anyway arbitrary and play no role in the following analysis). We compute S for the mid-spectrum eigenstates and for an ensemble of Haar random states, which serves as a benchmark. The probability

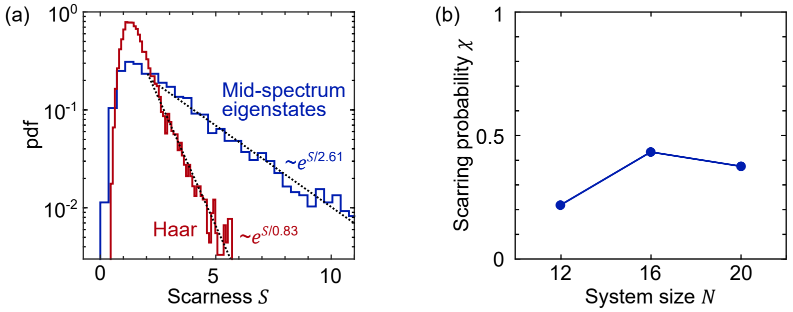

Fig. S3. **Scarness distribution and probability.** (a) We reproduce Fig. 3(b) from the main text for the probability distribution of the scarness S, but with logarithmic ordinate axis. This highlights an exponential scaling at large S, $\sim e^{S/S_0}$. From fits (dotted) we extract $S_0 = 0.83$ for the Haar random states, and $S_0 = 2.61$ for the eigenstates. (b) Scarring probability χ. While the number of accessible system sizes is limited, χ is shown to be rather large, around 40% for $N = 16$ and $N = 20$, and roughly constant (or at least not strongly decaying with N), consistently with the observed exponential scaling of $M_{\rm scarred}$ in the inset of Fig. 3(b). Here, we consider the same parameters as in Fig. 3(b).

distribution of the resulting S is shown in the main Fig. 3(b). While the mid-spectrum eigenstates of quantum chaotic Hamiltonians are often compared to Haar random states, and while the distributions obtained for the two indeed have some overlap, the distribution for the eigenstates is shifted towards larger values of S compared to that of the Haar random states. For instance, for $s = 1/2$, $N = 20$, and sector 0^+ we find that $\langle S \rangle_E - \langle S \rangle_H \approx 1.98\sqrt{\langle S^2 \rangle_H - \langle S \rangle_H^2}$, where $\langle \dots \rangle_E$ and $\langle \dots \rangle_H$ denote average over the ensembles of mid-spectrum eigenstates and Haar random states, respectively. That is, the average S for the eigenstates is two standard deviations larger than the average S for the Haar random states.

This mismatch can be used to quantify the number of scarred eigenstates. To this end we consider the M_e mid-spectrum eigenstates, say M'_e the number of them that have S larger than the 90-th percentile of the S for the Haar random states, and introduce the scarring probability χ as

$$\chi = \frac{M'_e}{M_e} - 0.1, \tag{S6}$$

where the -0.1 compensates for the fact that even for random Haar states S is larger than the 90-th percentile 10% of the times (by definition of 90-th percentile). If the eigenstates behaved like Haar random states, one would get $\chi = 0$. But because, instead, many of the eigenstates have S larger than most of the Haar random states, the scarring probability χ can then take relatively large values ($\sim 40\%$ for the considered parameters), indicating that a significant fraction of the eigenstates is scarred by the IS UPOs. Analogously, we can define the number of scarred mid-spectrum eigenstates as

$$M_{\rm scarred} = M'_e - 10\% M_e = \chi M_e, \tag{S7}$$

which we plotted in the inset of Fig. 3(b) in the main text.

Note that our analysis has focussed on the mid-spectrum eigenstates, the number of which, M_e, depends on how wide the central energy window is chosen, which is arbitrary. The $M_{\rm scarred}$ that we considered does not count the eigenstates outside of such energy window. Because the non-mid-spectrum eigenstates could also be scarred, one might say that $M_{\rm scarred}$ underestimates the number scarred eigenstates. At the end of the day, quantifying scarring is not an easy task, and there is no unique way of doing it. The analysis above succeeds in indicating that scarring affects many eigenstates, but for instance does not consider that certain eigenstates are scarred by multiple UPOs. There is thus room for a more systematic quantitative analysis of scarring in many-body systems, which goes however beyond the scope of this work.

Larger spins

In the main text we developed a general formalism valid for any spin length s, but tested it focusing on the most quantum case of $s = 1/2$. Here we show that a similar phenomenology of scarring emerges for larger spins $s = 1$, $3/2$ and 2. In Fig. S4 (in analogy with the main Fig. 2) we show that most of the mid-spectrum eigenstates have a projection Q that reflects the structure of the underlying UPOs. In Fig. S5(a) (in analogy with the main Fig. 4), we show that the structure of the eigenstates underpins memory effects in the time-averaged projection $\bar{Q}$. Note that, going deeper into the classical limit (i.e., increasing s), the features of the eigenstates and of the time-averaged projection become narrower, in accordance with the decreases of the effective $\hbar$ and in analogy with the single-particle case [1]. In Fig. S5(b,c) we show the scaling with s and N of the return

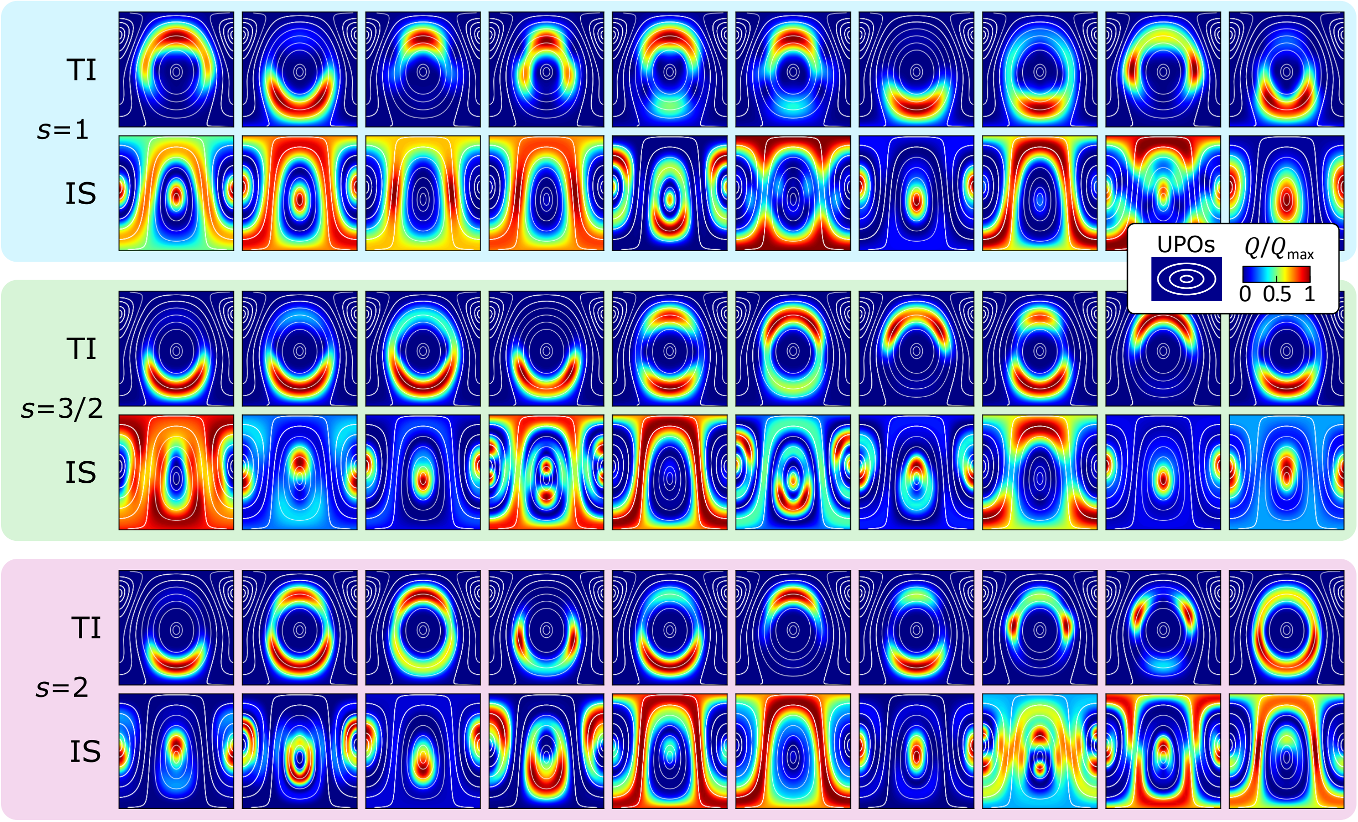

Fig. S4. **Quantum scars for larger spins.** Projection over the manifolds of TI and IS states of 10 consecutive eigenstates taken in the middle of the 0^+ symmetry sector for system size $N = 8$ and spin lengths $s = 1, 3/2$, and 2. Similar to the case of $s = 1/2$, also for larger spins s the eigenstates tend to reflect the structure of the underlying UPOs. Here, we considered the mixed-field Ising model with parameters as in Fig. 2 in the main text.

probability, $\bar{Q}(\mathbf{y} \to \mathbf{y})$, and of the ratio of return and cross probabilities, $\bar{Q}(\mathbf{y} \to \mathbf{y})/\bar{Q}(\mathbf{y} \to \boldsymbol{\mu})$. The return probability decays with both N and s, according to the growth of the size of the Hilbert space, $\sim (2s+1)^N$. Yet, for all simulated s the return probabilities remain clearly larger than the cross probabilities, by roughly four times, similar to Fig. 4(c) for spin $1/2$ in the main text.

II) ZERO-TH ORDER FLOQUET EXPANSION

In the frame precessing around $\boldsymbol{\mu}$ at the frequency of the IS UPOs, namely $\omega = \frac{2\pi}{T} = |\boldsymbol{\mu}|$, the Hamiltonian reads

$$\hat{H}_{\rm rot}(t) = \frac{1}{s}\sum_{i=1}^{N} \hat{\mathbf{s}}_i \mathbf{R}^{-1}(\omega t)\mathbf{J}\mathbf{R}(\omega t)\hat{\mathbf{s}}_{i+1}. \tag{S8}$$

The matrix $\mathbf{R}$ can be written using Rodrigues formula

$$\mathbf{R}(\omega t) = \mathbf{I} + \sin(\omega t)\mathbf{u}_\times + [1-\cos(\omega t)](\mathbf{u}_\times)^2, \tag{S9}$$

$$= \mathbf{u}\otimes\mathbf{u} + \sin(\omega t)\mathbf{u}_\times + \cos(\omega t)(\mathbf{I} - \mathbf{u}\otimes\mathbf{u}), \tag{S10}$$

where $\mathbf{u}_\times \mathbf{a} = \mathbf{u}\times\mathbf{a}$, $\mathbf{u} = \boldsymbol{\mu}/|\boldsymbol{\mu}|$, and where we used $\mathbf{I} + (\mathbf{u}_\times)^2 = \mathbf{u}\otimes\mathbf{u}$. The zero-th order Floquet expansion corresponds to substituting $\hat{H}_{\rm rot}(t)$ with its time average, $\hat{\bar{H}} = \frac{1}{T}\int_0^T dt \hat{H}_{\rm rot}(t)$. That is,

$$\hat{\bar{H}} = \frac{1}{s}\sum_{i=1}^{N} \hat{\mathbf{s}}_i \bar{\mathbf{J}}\hat{\mathbf{s}}_{i+1}, \tag{S11}$$

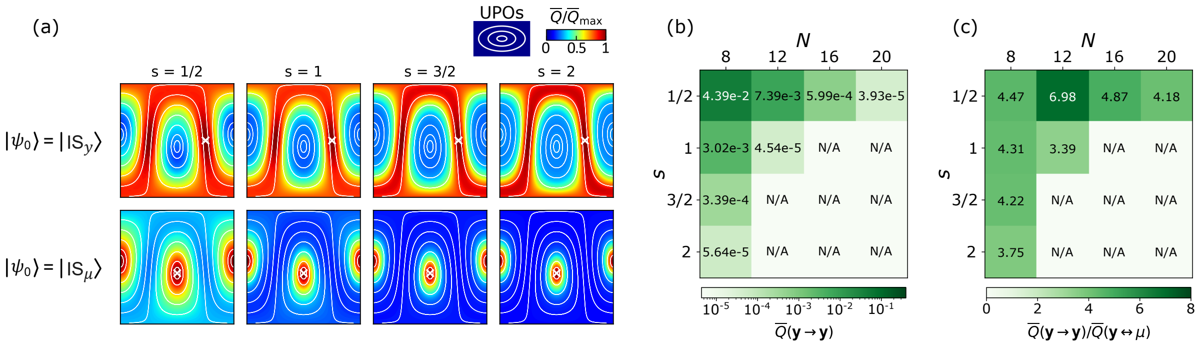

Fig. S5. **Weak ergodicity breaking for larger spins.** (a) Time-averaged projection $\bar{Q}$ over the manifold of IS states for $s = 1/2$, 1, 3/2, 2, and system size $N = 8$. The system is initialized on the IS UPOs aligned along $\mathbf{y}$ and $\boldsymbol{\mu}$ in the upper and lower rows, respectively, as marked by the white cross. For all considered s, the time-averaged projection is enhanced on the UPO the system is initialized on, that is, ergodicity is weakly broken. The regions of larger $\bar{Q}$ become increasing concentrated along the UPOs when increasing s. (b) Scaling of the return probability $\bar{Q}(\mathbf{y} \to \mathbf{y})$ with s and N. (c) Ratio of return probability $\bar{Q}(\mathbf{y} \to \mathbf{y})$ and cross probability $\bar{Q}(\mathbf{y} \to \boldsymbol{\mu})$ for various s and N (namely those for which exact diagonalization is amenable – the others are marked as N/A). The initial state is $|\psi_0\rangle = |\mathrm{IS}_y\rangle$ in all cases. The return probability remains around four times larger than the cross probability in all the cases we simulated. Here, we considered the mixed-field Ising model with parameters as in Fig. 2 in the main text.

with

$$\bar{\mathbf{J}} = \frac{1}{T}\int_0^T dt\, \mathbf{R}^{-1}(\omega t)\mathbf{J}\mathbf{R}(\omega t), \tag{S12}$$

$$= \int_0^1 dx \left[\mathbf{u}\otimes\mathbf{u}\mathbf{J}\mathbf{u}\otimes\mathbf{u} - \sin^2(2\pi x)\mathbf{u}_\times\mathbf{J}\mathbf{u}_\times + \cos^2(2\pi x)(\mathbf{I}-\mathbf{u}\otimes\mathbf{u})\mathbf{J}(\mathbf{I}-\mathbf{u}\otimes\mathbf{u})\right], \tag{S13}$$

$$= \mathbf{u}\otimes\mathbf{u}(\mathbf{u}\mathbf{J}\mathbf{u}) - \frac{1}{2}\mathbf{u}_\times\mathbf{J}\mathbf{u}_\times + \frac{1}{2}(\mathbf{I}-\mathbf{u}\otimes\mathbf{u})\mathbf{J}(\mathbf{I}-\mathbf{u}\otimes\mathbf{u}). \tag{S14}$$

Using the properties of the cross product we find

$$\mathbf{u}_\times\mathbf{J}\mathbf{u}_\times = (\mathbf{I}-\mathbf{u}\otimes\mathbf{u})(\mathbf{u}\mathbf{J}\mathbf{u} - \mathrm{Tr}\{\mathbf{J}\}) + (\mathbf{I}-\mathbf{u}\otimes\mathbf{u})\mathbf{J}(\mathbf{I}-\mathbf{u}\otimes\mathbf{u}), \tag{S15}$$

and thus

$$\bar{\mathbf{J}} = \mathbf{u}\otimes\mathbf{u}(\mathbf{u}\mathbf{J}\mathbf{u}) + \frac{1}{2}(\mathbf{u}\otimes\mathbf{u}-\mathbf{I})(\mathbf{u}\mathbf{J}\mathbf{u} - \mathrm{Tr}\{\mathbf{J}\}), \tag{S16}$$

$$= \mathbf{u}\otimes\mathbf{u}\frac{1}{2}(3\mathbf{u}\mathbf{J}\mathbf{u} - \mathrm{Tr}\{\mathbf{J}\}) - \mathbf{I}\frac{1}{2}(\mathbf{u}\mathbf{J}\mathbf{u} - \mathrm{Tr}\{\mathbf{J}\}). \tag{S17}$$

It will prove useful to further characterize the matrix $\bar{\mathbf{J}}$. Its eigenvectors are $\mathbf{u}$ and any two vectors orthogonal to it, with eigenvalues

$$\lambda_1 = \mathbf{u}\mathbf{J}\mathbf{u}, \qquad \lambda_2 = \lambda_3 = -\frac{1}{2}(\mathbf{u}\mathbf{J}\mathbf{u} - \mathrm{Tr}\{\mathbf{J}\}), \tag{S18}$$

and so

$$\det\left(\bar{\mathbf{J}}\right) = \frac{1}{4}(\mathbf{u}\mathbf{J}\mathbf{u})(\mathbf{u}\mathbf{J}\mathbf{u} - \mathrm{Tr}\{\mathbf{J}\})^2, \tag{S19}$$

$$\mathrm{Tr}\left\{\bar{\mathbf{J}}\right\} = \mathrm{Tr}\{\mathbf{J}\}. \tag{S20}$$

Moreover, we note that

$$\left[\mathbf{u}\otimes\mathbf{u}(3\mathbf{u}\mathbf{J}\mathbf{u} - \mathrm{Tr}\{\mathbf{J}\}) - 2(\mathbf{u}\mathbf{J}\mathbf{u})\mathbf{I}\right]\bar{\mathbf{J}} = (\mathbf{u}\mathbf{J}\mathbf{u})(\mathbf{u}\mathbf{J}\mathbf{u} - \mathrm{Tr}\{\mathbf{J}\})\mathbf{I}, \tag{S21}$$

and thus find the adjoint $\mathrm{Adj}(\bar{\mathbf{J}})$ such that $(\mathrm{Adj}(\bar{\mathbf{J}}))\bar{\mathbf{J}} = \det\left(\bar{\mathbf{J}}\right)\mathbf{I}$, namely

$$\mathrm{Adj}(\bar{\mathbf{J}}) = \frac{1}{4}(\mathbf{u}\mathbf{J}\mathbf{u} - \mathrm{Tr}\{\mathbf{J}\})\left[\mathbf{u}\otimes\mathbf{u}(3\mathbf{u}\mathbf{J}\mathbf{u} - \mathrm{Tr}\{\mathbf{J}\}) - 2(\mathbf{u}\mathbf{J}\mathbf{u})\mathbf{I}\right]. \tag{S22}$$

III) LYAPUNOV EXPONENT

We compute the classical Lyapunov exponent associated to the IS UPOs, in which the spin direction flips every other spin, $\{\mathbf{s}_j\} = (+\mathbf{s}, +\mathbf{s}, -\mathbf{s}, -\mathbf{s}, +\mathbf{s}, +\mathbf{s}, -\mathbf{s}, -\mathbf{s}, \dots)$. Various values of $\mathbf{s}$ correspond to various UPOs, and we should thus use $\mathbf{s}$ as a parameter, a tag of the considered UPO. Computing the Lyapunov exponent with respect to a periodic orbit can be complicated, but by moving to the frame precessing around the magnetic field $\boldsymbol{\mu}$, and assuming that $|\boldsymbol{\mu}|/|\mathbf{J}| \gg 1$, we transform the UPO into a fixed point of the dynamics, which considerably simplifies the computation of the Lyapunov exponent. In such frame the classical spin dynamics reads

$$\frac{d\mathbf{s}_i}{dt} = \left(\bar{\mathbf{J}}(\mathbf{s}_{i-1} + \mathbf{s}_{i+1})\right) \times \mathbf{s}_i. \tag{S23}$$

We want to study the evolution of small perturbation on top of the UPO. Let us say $\mathbf{s}_i = \nu_i \mathbf{s} + \boldsymbol{\epsilon}_i$, with $\{\nu_i\} = (+ + - - + + - - \dots)$ and $\boldsymbol{\epsilon}_i$ a small perturbation of order $\epsilon \ll 1$. Because $\nu_{i-1} + \nu_{i+1} = 0$, then $|\mathbf{s}_{i-1} + \mathbf{s}_{i+1}| \sim \epsilon$, and thus at first order in ϵ we can consider

$$\frac{d\boldsymbol{\epsilon}_i}{dt} \approx -\nu_i \mathbf{s} \times [\bar{\mathbf{J}}(\boldsymbol{\epsilon}_{i-1} + \boldsymbol{\epsilon}_{i+1})], \tag{S24}$$

$$= \nu_i \mathbf{M}(\boldsymbol{\epsilon}_{i-1} + \boldsymbol{\epsilon}_{i+1})], \tag{S25}$$

where $\mathbf{M} = -\mathbf{s}_\times \bar{\mathbf{J}}$. We perform a Fourier transformation

$$f_k = \sum_j e^{-ikj} f_j, \qquad f_j = \frac{1}{N} \sum_k e^{ikj} f_k, \tag{S26}$$

and, noting that $\nu_j = -\frac{1+i}{2} e^{i\pi/2j} - \frac{1-i}{2} e^{-i\pi/2j}$, get

$$(\nu_j(\boldsymbol{\epsilon}_{j-1} + \boldsymbol{\epsilon}_{j+1}))_k = \sum_j e^{-ikj} \nu_j (\boldsymbol{\epsilon}_{j-1} + \boldsymbol{\epsilon}_{j+1}), \tag{S27}$$

$$= -\sum_j e^{-ikj} \left(\frac{1+i}{2} e^{i\pi/2j} + \frac{1-i}{2} e^{-i\pi/2j} \right) (\boldsymbol{\epsilon}_{j-1} + \boldsymbol{\epsilon}_{j+1}), \tag{S28}$$

$$= -\sum_j \left(\frac{1+i}{2} e^{-i(k-\pi/2)j} + \frac{1-i}{2} e^{-i(k+\pi/2)j} \right) (\boldsymbol{\epsilon}_{j-1} + \boldsymbol{\epsilon}_{j+1}), \tag{S29}$$

$$= -(1+i) \cos\Big(k - \frac{\pi}{2}\Big) \boldsymbol{\epsilon}_{k-\pi/2} - (1-i) \cos\Big(k + \frac{\pi}{2}\Big) \boldsymbol{\epsilon}_{k+\pi/2}, \tag{S30}$$

$$= -(1+i) \sin(k) \boldsymbol{\epsilon}_{k-\pi/2} + (1-i) \sin(k) \boldsymbol{\epsilon}_{k+\pi/2}. \tag{S31}$$

The eigenproblem reads

$$\frac{d\boldsymbol{\epsilon}_k}{dt} = \lambda \boldsymbol{\epsilon}_k = -(1+i) \sin(k) \mathbf{M} \boldsymbol{\epsilon}_{k-\pi/2} + (1-i) \sin(k) \mathbf{M} \boldsymbol{\epsilon}_{k+\pi/2}, \tag{S32}$$

that is,

$$\lambda \begin{pmatrix} \boldsymbol{\epsilon}_k \\ \boldsymbol{\epsilon}_{k-\pi/2} \\ \boldsymbol{\epsilon}_{k+\pi/2} \\ \boldsymbol{\epsilon}_{k+\pi} \end{pmatrix} = \begin{pmatrix} 0 & -(1+i)\sin(k)\mathbf{M} & (1-i)\sin(k)\mathbf{M} & 0 \\ (1-i)\cos(k)\mathbf{M} & 0 & 0 & -(1+i)\cos(k)\mathbf{M} \\ (1+i)\cos(k)\mathbf{M} & 0 & 0 & (i-1)\cos(k)\mathbf{M} \\ 0 & (i-1)\sin(k)\mathbf{M} & (1+i)\sin(k)\mathbf{M} & 0 \end{pmatrix} \begin{pmatrix} \boldsymbol{\epsilon}_k \\ \boldsymbol{\epsilon}_{k-\pi/2} \\ \boldsymbol{\epsilon}_{k+\pi/2} \\ \boldsymbol{\epsilon}_{k+\pi} \end{pmatrix}. \tag{S33}$$

That is, we have obtained $N/4$ sets of 12-dimensional eigenproblems, for a total of $3N$ eigenvalues. Each eigenproblem is associated to a set of four momenta, namely $k, k - \frac{\pi}{2}, k + \frac{\pi}{2}$, and $k + \pi$. To label each set we can consider values of k up to $\frac{\pi}{2}$, namely $k = \frac{2\pi}{N} \times \left(1, 2, \dots, \frac{N}{4}\right)$, to which we will henceforth restrict. With the assistance of a computer, we find that the 12 eigenvalues of Eq. (S33) are $\left(0, 0, 0, 0, ae^{+i\frac{\pi}{4}}, ae^{+i\frac{\pi}{4}}, ae^{-i\frac{\pi}{4}}, ae^{-i\frac{\pi}{4}}, ae^{+i\frac{3\pi}{4}}, ae^{+i\frac{3\pi}{4}}, ae^{-i\frac{3\pi}{4}}, ae^{-i\frac{3\pi}{4}}\right)$, where a is some real positive number. To find it, we iterate Eq. (S32) once to get

$$\lambda^2 \boldsymbol{\epsilon}_k = -(1+i)\sin(k)\cos(k)\mathbf{M}^2 \left(-(1+i)\boldsymbol{\epsilon}_{k-\pi} + (1-i)\boldsymbol{\epsilon}_k\right) - (1-i)\sin(k)\cos(k)\mathbf{M}^2 \left(-(1+i)\boldsymbol{\epsilon}_k + (1-i)\boldsymbol{\epsilon}_{k+\pi}\right), \tag{S34}$$

$$= 2i\mathbf{M}^2 \sin(2k) \boldsymbol{\epsilon}_{k+\pi}, \tag{S35}$$

which we iterate again, to close the equation, getting

$$\lambda^4 \boldsymbol{\epsilon}_k = -4\mathbf{M}^4 \sin^2(2k)\boldsymbol{\epsilon}_k. \tag{S36}$$

Again with the help of a computer, we find that $\mathbf{M}^2$ has one vanishing eigenvalue and two degenerate eigenvalues $\alpha = -\mathbf{s}[\mathrm{adj}(\bar{\mathbf{J}})]\mathbf{s}$, thus getting $a = \sqrt{2|\alpha\sin(2k)|}$.

Putting all the results together, we thus find that the $3N$ eigenvalues of the problem are 0, with multiplicity N, and

$$\lambda_{k,m} = \sqrt{2|\alpha\sin(2k)|}e^{i\frac{\pi}{4}+im\frac{\pi}{2}}, \tag{S37}$$

with multiplicity 2 and for $m = 1, 2, 3, 4$ and for $k = \frac{2\pi}{N} \times \left(1, 2, \ldots, \frac{N}{4}\right)$. Note: the fact that N of the $3N$ eigenvalues vanish is simply due to the fact that the three components of the spins are not independent, but constrained by $|\mathbf{s}_i|^2 = 1$. Note as well that for $N = 4$ all the Lyapunov exponents vanish, $\lambda_{k,m} = 0$, consistently with Ref. [2]. Here, we should focus on $N \geq 8$.

The instability exponent λ is the largest of the real parts of the eigenvalues, obtained for $k = \frac{\pi}{4}$ and giving $\lambda^2 = |\alpha| = |\mathbf{s}[\mathrm{adj}(\bar{\mathbf{J}})]\mathbf{s}|$. Using the expression found in Eq. (S22) we get

$$\lambda^2 = \frac{1}{4}\left|(\mathbf{uJu} - \mathrm{Tr}\{\mathbf{J}\})\left[(\mathbf{u}\cdot\mathbf{s})^2(3\mathbf{uJu} - \mathrm{Tr}\{\mathbf{J}\}) - 2\mathbf{uJu}\right]\right|. \tag{S38}$$

For instance, for the Ising model in a field we get

$$\lambda^2 = \frac{1}{4}J^2u_x^2\left[(1-3u_z^2)(\mathbf{u}\cdot\mathbf{s})^2 + 2u_z^2\right]. \tag{S39}$$

Note, the fact that λ in Eq. (S38) does not depend on N is not obvious a priori: it is a special feature of the considered IS UPOs, for which the dynamics decouples for groups of just four momenta in Eq. (S33).

IV) CLASSICAL FIDELITY

The overlap of a classical state $|\{\mathbf{s}'_j\}\rangle$ onto a point $\{\mathbf{s}_j\}$ of the classical phase space reads

$$Q = \left|\langle\{\mathbf{s}_j\}|\{\mathbf{s}'_j\}\rangle\right|^2 = \prod_j \left|\langle\mathbf{s}_j|\mathbf{s}'_j\rangle\right|^2 = \prod_j \left(\frac{1+\mathbf{s}'_j\cdot\mathbf{s}_j}{2}\right)^{2s}. \tag{S40}$$

We note in passing that $Q \approx \exp\left(-\frac{s}{2}\sum_j \theta_j^2\right)$, with θ_j the angle between $\mathbf{s}_j$ and $\mathbf{s}'_j$ and where the approximation becomes more accurate when increasing s. Indeed, the states $|\{\mathbf{s}_j\}\rangle$ are the natural generalization of Gaussian states for spins.

Consider a point $\{\mathbf{s}_j\}$ of the classical phase space. A perturbation of this point is obtained by rotating each spin by an angle θ_j drawn at random from a Gaussian distribution with standard deviation $\Delta = 10^{-5}$ (almost identical results are obtained for any $\Delta \ll 1$, provided that the time averaging is performed over a time $\gg \frac{1}{\lambda}\log\Delta$). Considering many such perturbations we obtain an ensemble of phase-space points $\{\mathbf{s}_j^{(r)}\}$, with $r = 1, 2, \ldots, R$, that are closely localized around the reference point $\{\mathbf{s}_j\}$. By classically time evolving the ensemble, we obtain the corresponding classical time-averaged projection

$$\bar{Q}_c(\{\mathbf{s}_j\}) = \frac{1}{R}\sum_{r=1}^{R}\lim_{t\to\infty}\frac{1}{t}\int_0^t d\tau \prod_j\left(\frac{1+\mathbf{s}_j\cdot\mathbf{s}_j^{(r)}(t)}{2}\right)^{2s}. \tag{S41}$$

Note that $\bar{Q}_c(\{\mathbf{s}_j\})$ is exponentially small in both N and s. The parameter s controls how heavily a misalignment between $\mathbf{s}_j^{(r)}$ and $\mathbf{s}_j$ is penalized. The larger s, the more unlikely that a trajectory will meaningfully contribute to the sum, and the larger the number of samples R required for convergence. In Fig. 4 we consider $s = 1/2$, for which a good convergence is obtained for $R = 2\times10^5$ samples. This choice is ultimately arbitrary, and we expect that the same key finding (that $\bar{Q}_c$ does not depend on the initial condition) would be found for larger values of s, although at the cost of a larger computational burden.

[1] E. J. Heller, Bound-state eigenfunctions of classically chaotic hamiltonian systems: scars of periodic orbits, Physical Review Letters **53**, 1515 (1984).

[2] R. Steinigeweg and H.-J. Schmidt, Heisenberg-integrable spin systems, Mathematical Physics, Analysis and Geometry **12**, 19 (2009).
